# Second harmonic generation in the bulk of silicon induced by an electric field of a high power terahertz pulse

**DOI:** 10.1038/s41598-019-46284-8

**Published:** 2019-07-05

**Authors:** A. V. Ovchinnikov, O. V. Chefonov, E. D. Mishina, M. B. Agranat

**Affiliations:** 1 Joint Institute for High Temperatures of the Russian Academy of Sciences (JIHT RAS), Izhorskaya 13 Bldg. 2, Moscow, 125412 Russian Federation; 2 MIREA - Moscow Technological University, Vernadsky Ave. 78, Moscow, 119454 Russian Federation

**Keywords:** Ultrafast photonics, Semiconductors, Electronic properties and materials, Nonlinear optics

## Abstract

The experimental findings on the second harmonic generation (SHG) in centrosymmetric crystal silicon are reported. The SHG is induced by extremely high electric field (up to 15 MV/cm) parallel to the crystal surface of a short terahertz (THz) pulse while probing by an infrared femtosecond optical pulse. The SHG under such unique conditions is reported for the first time. At the electric field amplitude above 8 MV/cm, the quadratic dependence of the SHG yield integrated over the THz pulse duration on the electric field is violated and SHG yield is not changed with a further increase of the THz field. Saturation of SHG intensity at high electric fields is explained in terms of carrier density increase due to impact ionization and destructive interference of electric-field induced and current induced nonlinear polarizations.

## Introduction

Second harmonic generation has been used for many years for studying properties of bulk and thin film materials^1^. SHG has been proven to be a most sensitive probe of electric and magnetic symmetries as well as their changes to external fields^2^. High sensitivity and selectivity of SHG to the bulk and interface properties^3^ can even exceed the capabilities of existing techniques. Thus, an important issue is the SHG efficiency for detecting high-frequency coherent transient processes caused by electrical and/or magnetic fields of low-cycle THz pulses. Investigation in this direction will enable one to develop new nonlinear optical methods for studying properties of semiconductor materials and nanostructures.

Detection of freely propagating THz radiation by use of optical second-harmonic generation in silicon was reported earlier^4^. Being attractive for centrosymmetric media, this approach allows to extend the class of materials to be used in electro-optical sampling measurements. Previously performed works on the study of the THz induced SHG were carried out at electric field strengths less than 1 MV/cm^4–10^.

In this study for the first time we present the findings on effect of high electric field strength of a THz pulse (up to 15 MV/cm) on bulk second-order susceptibility in silicon by use of second harmonic generation of an infrared femtosecond laser pulse propagating through a wafer.

## Results

The used in the experiments silicon sample with low electron density (as a p-type) is non-absorbing also for THz radiation, in contrast to n-type silicon with a high electron density^11,12^.

The transmittance measurement of silicon wafer depending on the electric field of the THz pulse incident on the sample were carried out in order to accurately interpret the experimental findings and to estimate the electric field strength of the THz pulse at the backside of the silicon wafer (this field serves as a pump for SHG, see Methods).

The electric field strength of the incident THz pulse was changed by adjusting the laser pump energy incident on the THz crystal. The transmittance was determined as a ratio of the THz pulse energy transmitted through the silicon sample to the THz pulse energy incident on the sample.

**Figure 1:**
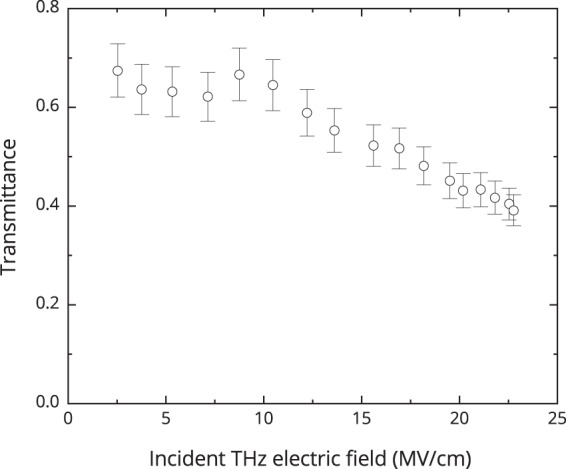
Transmittance of silicon as a function of the electric field strength of the THz pulse incident on the sample.

The transmittance of the silicon wafer considering the Fresnel reflection and absorption losses is shown in Fig. 1. One can see that the transmittance stays nearly the same at electric field strengths in the range from 2 to 8 MV/cm. However, with increasing THz electric field strength above 10 MV/cm the transmittance of silicon begins to decrease up to 40% at 22 MV/cm.

In addition, the measured dependence of THz radiation reflectivity on its electric field strengths did not show any changes. Therefore, it might be supposed that a decrease in transmittance is affected solely by an increase in absorption.

On the assumption that changes in absorption are due to impact ionization and the following increase in free carrier density, the estimated at 1 THz in the frame of the Drude model electron density does not exceed 10^16^ cm^−3^.

As at this concentration the plasma frequency is much lower the optical probe radiation frequency, no significant changes in dielectric function and coherence length for radiation at 1240 and 620 nm occur.

**Figure 2:**
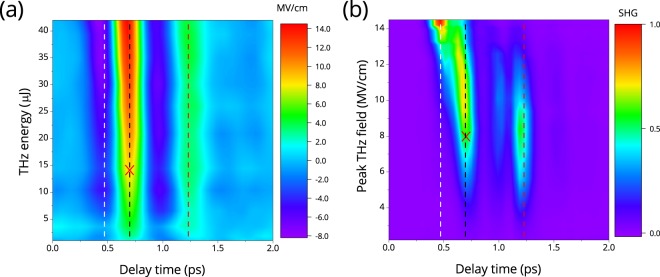
(**a**) Temporal profile of electric field of transmitted THz pulse as a function of its energy; (**b**) Dependence of second harmonic intensity on time (x-axis) and electric field strength of THz pulse. Dashed lines correspond to to minima and maxima in THz field oscillations at: *t*_*del*_ = 0.47 ps (white), *t*_*del*_ = 0.7 ps (black) and *t*_*del*_ = 1.23 ps (red); the crosses correspond to transient field *E*_Ω_ = 8 MV/cm.

Contour maps of the electric field temporal profile of the transmitted THz pulse as a function of its energy and the temporal profile of the second harmonic radiation during THz irradiation as a function of the THz electric field strength are shown in Fig. 2. The SHG measurements were carried out at a constant energy of the 100 fs laser pulse of 1 *μ*J that corresponded to intensity of 0.5 · 10^12^ W/cm^2^.

The electric field waveforms of the transmitted THz pulse (Fig. 2(a)) were measured using the conventional electro-optical sampling (EOS) method in GaP crystal, a detailed description of the setup is presented in^12^.

As follows from the experimental results, the temporal profiles of the electric field and the second harmonic intensity are very different. Really, the input THz pulse consists mainly of large positive maximum at t = 0.7 ps surrounded by two negative maxima with approximately 3 times lower amplitude at t = 0.4 and 1 ps. The profile does not change much (consists of parallel lines in Fig. 2(a)) while propagating through the sample. This means that we have not observed strong self-influence of the THz pulse. For SHG, situation is different. Although for the lowest THz field, the main maxima positions of THz field and SHG intensity are quite close, increase of THz field results in a shift on a time scale of main SHG maximum towards the first (small and negative) maximum of THz field. Quantitative analysis of these dependences is quite difficult because SHG intensity and THz field are measured by different techniques, both with their own zeroes on a delay time scale. Dynamics of the second harmonic radiation and its correlation with the THz radiation waveform is a subject of separate scientific research. In the further analysis, the second harmonic radiation energy integrated over the whole pulse will be considered as function of electric field amplitude of the THz pulse.

**Figure 3:**
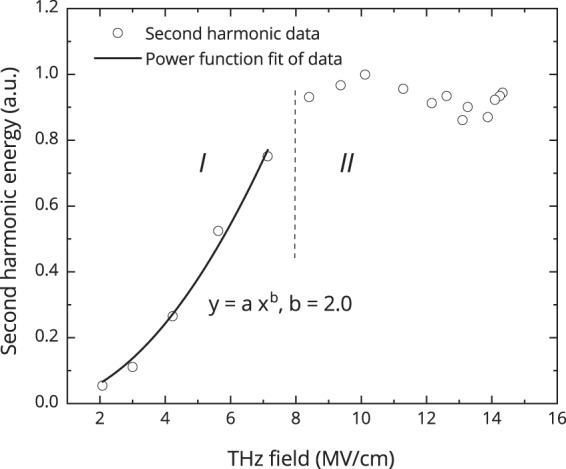
The dependence of the second harmonic radiation energy coming out from the sample on the electric field strength of the THz pulse.

The dependence of the second harmonic radiation energy coming out from the sample on the electric-field strength of the THz pulse is shown in Fig. 3, the values are obtained through integrating of the second harmonic intensity temporal profiles over time in the 0–2 ps range (Fig. 2(b)). The electric field strength of the THz pulse in Fig. 3 was determined as product of incident electric field strength and the corresponding transmittance coefficient from Fig. 1.

As it could be seen from the graph, the dependence of the second harmonic radiation energy is well approximated by a second-order power law in the electric field strength range from 2 to 8 MV/cm. However, at higher fields in the 8–14 MV/cm range the second harmonic radiation yield appears to be invariable on the THz pulse electric field. As one can see in Fig. 1, the transmittance of silicon also changes in the same range.

## Discussion

For a centrosymmetric crystal like silicon the even-order bulk dipole nonlinear susceptibility tensors are zero, and bulk dipole SHG is absent. In the absence of external factors that could break symmetry (electric field or mechanical deformation) there is a weak contribution to a SHG signal attributed to quadrupole effects. A symmetry could be also broken at the interface in a thin near-surface layer of several interatomic distances in thickness that also has second-order nonlinear susceptibility^13–15^. However, bulk quadrupole effects are small in comparison to bulk dipole contribution^16^ that we consider in this paper. In the experiments we did not observe the SHG signal in the absence of the THz field, or that signal was lower the detector sensitivity.

When applying a DC electric field to a silicon sample the SHG is determined by the third order nonlinear susceptibility that is referred to as an electric field-induced second harmonic (EFISH)^17,18^ or TEFISH if an electric field of a THz pulse is applied^4–10^, the corresponding nonlinear polarization has the form:

1 $$P(2\omega )\propto {\chi }^{(3)}{E}_{{\rm{\Omega }}}{E}_{\omega }{E}_{\omega },$$

where ${\chi }^{(3)}$ is the phenomenological tensor of the bulk dipole cubic susceptibility of the medium, which does not vanish in centrosymmetric media, ${E}_{\omega }$ and ${E}_{{\rm{\Omega }}}$ are electric fields of the infrared laser and of the THz pulses, respectively, both taken inside a material.

For noncentrosymmetric medium, nonlinear polarization is written as $P(2\omega )={\chi }^{(2)}{E}_{\omega }{E}_{\omega }$ with ${\chi }^{(2)}={\chi }^{(2)}({E}_{{\rm{\Omega }}})$ being dependent on electric field ${E}_{\omega }$ of THz pulse. Phenomenologically for low enough electric field (in our experiments the ratio ${E}_{{\rm{\Omega }}}\ll {E}_{at}={10}^{8}-{10}^{10}\,{\rm{V}}/{\rm{cm}}$ is valid), the function can be expended in Taylor series and the first term gives linear dependence of polarization (equation 1) on THz electric field and corresponding quadratic dependence of SHG intensity. It should be emphasized that both bound and free electrons may give contribution to ${\chi }^{(3)}$ in semiconductors^19^.

With an external field increase, linear dependence may violate quadratic law by different effects. Such effects have been extensively studied since 80-th, in semiconductors as well as in other materials at optical frequencies for second and third harmonics generation, sum-frequency generation and optical Kerr effect. Both bound and free electrons may give contribution to ${\chi }^{(3)}$ in semiconductors^19,20^, the latter providing dependences of ${\chi }^{(3)}$ on carrier density. For an electric field at THz frequencies, quasi-static conditions with high electric field application can be considered.

As shown in^11,21–24^ high electric fields lead to the effect of impact ionization that in turn results in a short-time occupation by electrons of conduction band and electron-hole pairs generation. Electrons and holes moving in a field of a THz pulse create a transient current that holds only during the action of a THz pulse. As presented in^13,25^ current in a semiconductor is a source of nonlinearity due to arising asymmetry in occupation of valence band and the dependence of the nonlinear susceptibility on the field becomes dependent on current density: ${\chi }^{(2)}({E}_{{\rm{\Omega }}})={\chi }_{j}^{(2)}(j({E}_{{\rm{\Omega }}}))$.

Analogously to^13,25^, consider linear dependence of a second-order nonlinear susceptibility on a current density. Then we can put the total susceptibility as a sum of two contributions: pure field dependent and current dependent terms: ${\chi }^{(2)}({E}_{{\rm{\Omega }}})+{\chi }_{j}^{(2)}(j({E}_{{\rm{\Omega }}}))={\chi }^{(3)}{E}_{{\rm{\Omega }}}+{\chi }^{(3)}j({E}_{{\rm{\Omega }}})$. For electric field lower than a threshold value for impact ionization, ${E}_{{\rm{\Omega }},th}$ the first term exists only. For ${E}_{{\rm{\Omega }}} > {E}_{{\rm{\Omega }},th}$, the second term joins the game. Estimations for these two terms are required in order to justify the suggested mechanisms.

For estimating the current density, expression $j=e{n}_{e}{\nu }_{e}+e{n}_{h}{\nu }_{h}$ can be used where *n*_*e*_, *n*_*h*_ and ${\nu }_{e}$, ${\nu }_{h}$ are electron and hole concentrations and velocities, respectively. Here ${n}_{e}={10}^{5}$, ${n}_{h}={10}^{15}\,{{\rm{cm}}}^{-3}$ (initial electron and hole concentrations in the used p-type silicon). At high electric fields of the THz pulse free carrier concentration increases due to impact ionization and reaches ${n}_{e}={n}_{h}=3\cdot {10}^{15}\,{{\rm{cm}}}^{-3}$ at ${E}_{{\rm{\Omega }}}$ = 14 MV/cm. The estimation of free carrier concentration was obtained from the experimental results (Fig. 1) using changes in absorption of the THz pulse. Let us compare a ratio of field and current contributions into overall nonlinear susceptibility. For 1 ps, while the THz pulse lasts, and even more so for 0.3 ps with a field of one sign, the electron motion can be considered ballistic. In the expression for current density *j* free carrier velocity ${\nu }_{e,h}$ is determined through electron and hole mobilities ${\mu }_{e,h}$ and an electric field strength of the THz pulse ${E}_{{\rm{\Omega }}}$: ${\nu }_{e,h}={\mu }_{e,h}\,{E}_{{\rm{\Omega }}}$. For estimations we used *μ*_*e*_ = 1400 cm^2^/(V s), *μ*_*h*_ = 450 cm^2^/(V s)^26^. For the electric field strength of 2 MV/cm (in the region of quadratic dependence of second harmonic yield on the electric field strength) current density is *j* = 1.4 · 10^5^ A/cm^2^, and for 14 MV/cm (in the saturation region) *j* = 1.2 · 10^7^ A/cm^2^.

In DC experiments, current-induced nonlinear polarization in silicon is $${\chi }^{(2)}(j={10}^{3}\,A/c{m}^{2})=3\cdot {10}^{-15}\,{\rm{m}}/{\rm{V}}$$^13^ and $${\chi }^{(2)}(j={10}^{4}\,A/c{m}^{2})={10}^{-14}\,{\rm{m}}/{\rm{V}}$$^25^. Then rough estimation in assumption of linear approximation gives for our experiment at 2 MV/cm ($$j=1.4\cdot {10}^{5}\,A/c{m}^{2}$$) $${\chi }^{(2)}(j)=1.4\cdot {10}^{-13}\,{\rm{m}}/{\rm{V}}$$, and at 14 MV/cm ($$j=1.2\cdot {10}^{7}\,A/c{m}^{2}$$) – $${\chi }^{(2)}(j)=1.2\cdot {10}^{-11}\,{\rm{m}}/{\rm{V}}$$. For field-induced nonlinear susceptibility $${\chi }^{(2)}({E}_{{\rm{\Omega }}})={\chi }^{(3)}{E}_{{\rm{\Omega }}}$$. For silicon, $${\chi }^{(3)}=0.5\cdot {10}^{-19}\,{{\rm{m}}}^{2}/{{\rm{V}}}^{2}$$ at 1.2 *μ*m^27^ and then at 2 MV/cm $${\chi }^{(2)}({E}_{{\rm{\Omega }}})={10}^{-11}\,{\rm{m}}/{\rm{V}}$$, and at 14 MV/cm – $${\chi }^{(2)}({E}_{{\rm{\Omega }}})=$$$$7\cdot {10}^{-11}\,{\rm{m}}/{\rm{V}}$$. Thus in this estimation for lower THz field, electric-field induced term substantially dominates over the current-induced term. With the further THz power increase, the current-induced term increases much faster due to impact ionization and reaches the same order of magnitude as the electric-field induced term.

The presence of two contributions probably can explain saturation of SH intensity at high electric fields in terms of destructive interference of electric-field induced and current induced nonlinear polarizations. It is also important to note that field-induced nonlinearity is connected mostly with bound electrons, which concentration is not changed during the impact ionization process, because it is is much higher (10^22^ cm^−3^) than free electron density.

From the point of view of polarization and current directions, it is worth noting that a condition of optimal geometry is fulfilled by intrinsic current nature which is collinear to the driving field. The experimentally observed saturation of second harmonic intensity indicates the factors confining carrier density and current under the high carrier density and high currents induced by the impact ionization.

The suggested approach allows qualitative explanation of SHG temporal-field behavior coded in Fig. 2. Initially for low fields, the simple scenario of EFISH takes place. At the main maximum of the THz oscillation, the field increase up to 8 MV/cm (*t*_*del*_ = 0.7 ps, black dashed line below the cross) results in correspondent increase of SHG. The same is true for the first oscillation of THz field (“negative maximum“ at *t*_*del*_ = 0.47 ps, white line). For higher fields, impact ionization occurs, a current-induced SHG comes to play and the SHG intensity decreases (see right panel, black line, above the cross). The current-induced SHG field is apparently shifted in phase regarding to the EFISH phase in a way providing the total SHG signal decrease. For larger time delay, such a simple picture does not work: for *t*_*del*_ = 1.23 ps the SHG signal first increases but then starts to decrease at much lower field of about 3–4 MV/cm. This might be due to some cumulative effects which require consideration of precise impact ionization model.

## Conclusions

In this paper, for the first time, the results are reported of experimental studies in a bulk of p-type silicon crystal of a second harmonic generation of a femtosecond chromium-forsterite laser radiation induced by a strong electric field (up to 15 MV/cm) of a THz pulse. It is shown that the dependence of the second harmonic yield integrated over the THz pulse duration on the pulse electric field is quadratic in the range up to 8 MV/cm. It shown, for the first time, that the second harmonic yield does not depend on the THz field amplitude in the range of 8 MV/cm to 15 MV/cm, and the temporal shape of the of the second harmonic intensity does not correlate with the temporal shape of the THz pulse. In the same range, a decrease in the THz radiation transmission in a silicon sample is observed. It is assumed that the experimentally observed saturation of the SH output is due to the short-term population of the conduction band with electrons and the generation of electron-hole pairs as a result of impact ionization. Electron-hole pairs in the field of THz waves, create a transient current, which is the source of nonlinearity. The saturation of the SHG yield in strong electric fields can be explained in terms of the destructive interference of nonlinear polarizations caused by the electric field and current.

## Methods

**Figure 4:**
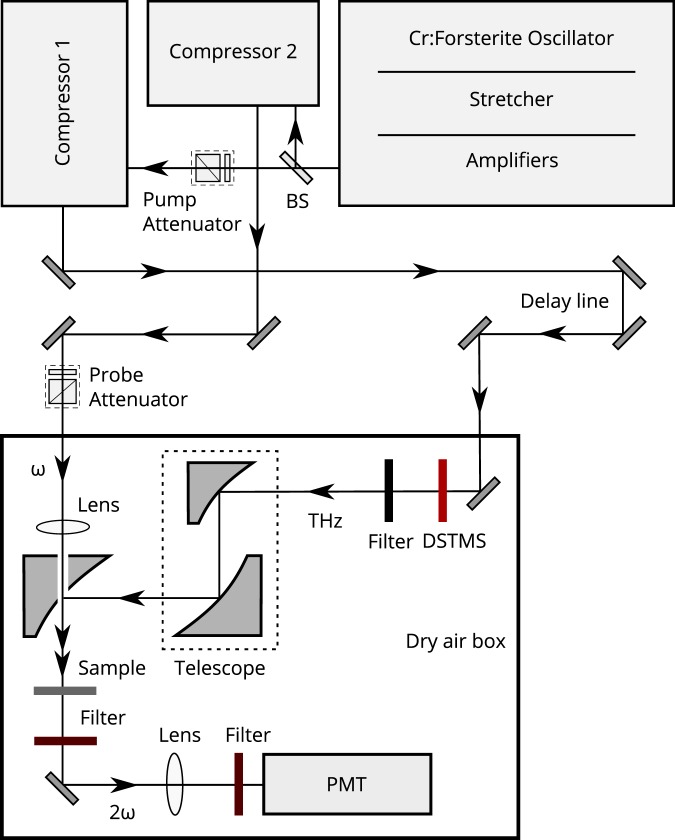
Experimental scheme of second harmonic generation and monitoring in p-type silicon under the action of electric field of terahertz pulsed radiation. BS — beam-splitter of laser radiation, PMT — photomultiplier tube.

An unique Cr:forsterite femtosecond laser system that provides 100-fs laser pulses at a central wavelength of 1240 nm with above 40 mJ pulse energy and at 10 Hz repetition rate was used in the experiments^28^. High power THz pulses could be generated with a high efficiency using this laser system^29,30^. An experimental scheme for studying high-power terahertz electric field induced second harmonic generation in silicon at 620 nm is shown in Fig. 4.

The terahertz beam was expanded with a telescope and then focused onto a sample to a 260-*μ*m spot (at a level of 1/e^2^)^31,32^ using a 90° off-axis parabolic mirror with a focus length of 50.8 mm and a diameter of 50.8 mm. Terahertz pulse energy at a focal plane was measured by means of a calibrated Golay cell resulting in 106 ± 10 *μ*J that corresponds to the incident electric field strength of 22 ± 1 MV/cm. The estimation of the electric field strength was made in accordance with the Poynting’s theorem stating the dependence between the electric field amplitude and time-averaged intensity of an electromagnetic wave $I=S=\langle c{\varepsilon }_{0}{E}^{2}\rangle$. A more detailed description of the estimation and verification of a THz electric field strength value is presented in our previous studies^11,12,31,32^.

The time-delayed probe beam at 1240 nm coaxially propagated with the THz pump and using a lens was focused through an aperture of the off-axis parabola to a spot of 50 *μ*m in diameter.

In the experiments we utilized THz radiation with linear polarization parallel to that of the probe radiation. The THz and probe beams were incident normally to the sample surface. This geometry is optimal for conversion of the probe radiation into the second harmonic during the action of the THz field, as other electro-optic effects^33^.

Polarized attenuators consisted of the Glan-Tompson prism and a half-wave plate were used to adjust the energy of the optical probe and the THz crystal pump pulses (THz energy pulse control).

The second harmonic radiation at 620 nm coming out from the sample’s backside was collimated with a positive lens and then recorded using the photomultiplier tube. The laser radiation at 1240 nm transmitted through the sample was attenuated by means of two narrow-band interference filters at 620 nm with an overall attenuation coefficient of 10^10^.

Experiments were carried with a polished p-doped Si wafer 245 *μ*m thick with a crystallographic orientation (100), free carrier density of 1.6 · 10^15^ cm^−3^ and mobility of 325 cm^2^/(V · s) (from the Hall effect measurements).

A distinctive feature of the experiments on optical second harmonic generation is related with the use of the 1240 nm (0.98 eV) laser radiation for which silicon with a band-gap energy (1.1 eV) is transparent. At the same time, in silicon the 620 nm second harmonic radiation decays with a penetration depth of ${l}_{{\mathtt{penetr}}}\simeq 2\,\mu$m^34^, while a coherence length ${L}_{{\mathtt{c}}}=\lambda$/$(4\pi {\rm{\Delta }}n)$, being a characteristic length of a maximum energy transfer from fundamental frequency to second harmonic, and vice versa, is about 0.8 *μ*m.
